# Structural insights into human CCAN complex assembled onto DNA

**DOI:** 10.1038/s41421-022-00439-6

**Published:** 2022-09-09

**Authors:** Tian Tian, Lili Chen, Zhen Dou, Zhisen Yang, Xinjiao Gao, Xiao Yuan, Chengliang Wang, Ran Liu, Zuojun Shen, Ping Gui, Maikun Teng, Xianlei Meng, Donald L. Hill, Lin Li, Xuan Zhang, Xing Liu, Linfeng Sun, Jianye Zang, Xuebiao Yao

**Affiliations:** 1 MOE Key Laboratory for Cellular Dynamics, the First Affiliated Hospital, CAS Center for Excellence in Biomacromolecules, and School of Life Sciences, University of Science and Technology of China, Hefei, China; 2 Anhui Key Laboratory for Cellular Dynamics and Chemical Biology, Hefei, China; 3 Comprehensive Cancer Center, University of Alabama, Birmingham, AL USA; 4 CAS Center for Excellence in Molecular and Cell Sciences, Shanghai, China

**Keywords:** Mitosis, Cryoelectron microscopy

## Abstract

In mitosis, accurate chromosome segregation depends on kinetochores that connect centromeric chromatin to spindle microtubules. The centromeres of budding yeast, which are relatively simple, are connected to individual microtubules via a kinetochore constitutive centromere associated network (CCAN). However, the complex centromeres of human chromosomes comprise millions of DNA base pairs and attach to multiple microtubules. Here, by use of cryo-electron microscopy and functional analyses, we reveal the molecular basis of how human CCAN interacts with duplex DNA and facilitates accurate chromosome segregation. The overall structure relates to the cooperative interactions and interdependency of the constituent sub-complexes of the CCAN. The duplex DNA is topologically entrapped by human CCAN. Further, CENP-N does not bind to the RG-loop of CENP-A but to DNA in the CCAN complex. The DNA binding activity is essential for CENP-LN localization to centromere and chromosome segregation during mitosis. Thus, these analyses provide new insights into mechanisms of action underlying kinetochore assembly and function in mitosis.

## Introduction

Correct chromosome segregation relies on the centromere, a specialized chromatin domain present throughout the cell cycle that acts as a platform on which transient assembly of the kinetochore occurs during mitosis^1,2^. In eukaryotic cells, kinetochores, which are sophisticated molecular machines for cell division fate decisions, constitute a dynamic link between kinetochore microtubule attachment and spindle checkpoints, a signaling cascade that prevents chromosome segregation before completion of bi-orientation during cell division. In fact, early electron microscopic (EM) analyses have indicated that the morphology of the outer kinetochore structure changes dynamically during mitotic progression^3,4^, suggesting that the kinetochore is dynamically assembled and functionally matured in mitosis. However, the molecular details underlying kinetochore assembly and its functional coupling to chromosome movements remain less characterized.

The CENP-A nucleosome (CENP-A^Nuc^) is surrounded by a group of 16 proteins, the constitutive centromere-associated network (CCAN) and organized in various stable subcomplexes^5–7^. CENP-C and another CCAN subunit, CENP-T, provide a platform for assembly of the outer layer of kinetochores^7,8^. The outer kinetochore comprises three subcomplexes, KNL1, MIS12, and NDC80, which are collectively referred to as the KMN network^9^. In addition to its regulatory functions, the KMN network, through its NDC80 complex, provides a site for accurate segregation of chromosomes to the daughter cells^10,11^. Unlike yeast, in which the molecular architecture of the kinetochore has been extensively defined^12–14^, the molecular architecture of metazoan CCANs and how multiple CCAN modules constitute a functional human kinetochore are less clear as the regional kinetochores of metazoans are generated from multiple copies of the CCAN and KMN to project large disk-like structures^15^. In particular, how CCAN components are organized and integrated with centromeric nucleosome and the KMN network for spindle microtubule interactions in mitosis remain unclear^16^.

Since metazoans have evolved elaborate regional centromere and spindle checkpoint machinery to ensure faithful chromosome segregation in mitosis^17,18^, we sought to delineate the molecular architecture of human CCAN and to perform a structural comparison of kinetochores between yeast and humans by conducting cryo-electron microscopy (cryo-EM) analysis of a human CCAN complex reconstituted in vitro with purified components. Interestingly, CENP-LN in CCAN complex forms a channel-like structure that binds to centromeric linker DNA. Our structure and biochemical analyses revealed a surprising link between CENP-LN binding to DNA in the context of CCAN and their functional relevance during mitotic chromosome segregation in mitosis.

## Results

### Structure of the human CCAN complex

To understand the molecular basis underlying human kinetochore organization, we derived the structure of the CCAN complex using a cryo-EM single particle analysis. Human CCAN is composed by five modules of CENP-C, CENP-LN, CENP-HIKM, CENP-OPQUR and CENP-TWSX. We reconstituted a 16-subunit human CCAN complex assembled on CENP-A^Nuc^ wrapped with 147 bp of DNA using the Widom 601 sequence (Supplementary Fig. S1a–g). Initial 3D classification and refinement resulted in an EM map with an overall resolution at 3.3 Å of CCAN complex, estimated by the gold-standard Fourier Shell Correlation method (Supplementary Fig. S2a–f). Further classifications and reconstructions gave two maps with distinct features in an interior tunnel, representing the CCAN complex alone (apo-CCAN) and CCAN in complex with a fragment of DNA (CCAN-DNA) (Supplementary Fig. S2f). However, no particles corresponding to the CCAN-CENP-A^Nuc^ complex were observed. The 3.3 Å map was of high quality and was used for model building (Supplementary Fig. S2a–f, Table S1). Individual models of CCAN subunits were either solved experimentally or predicted by AlphaFold2 (AF2)^19^ and the I-TASSER program was used to facilitate model building^20^. The built model was then docked into the EM map of CCAN–DNA complex at 3.7 Å and further refined (Supplementary Fig. S2e, f, Table S1).

**Fig. 1:**
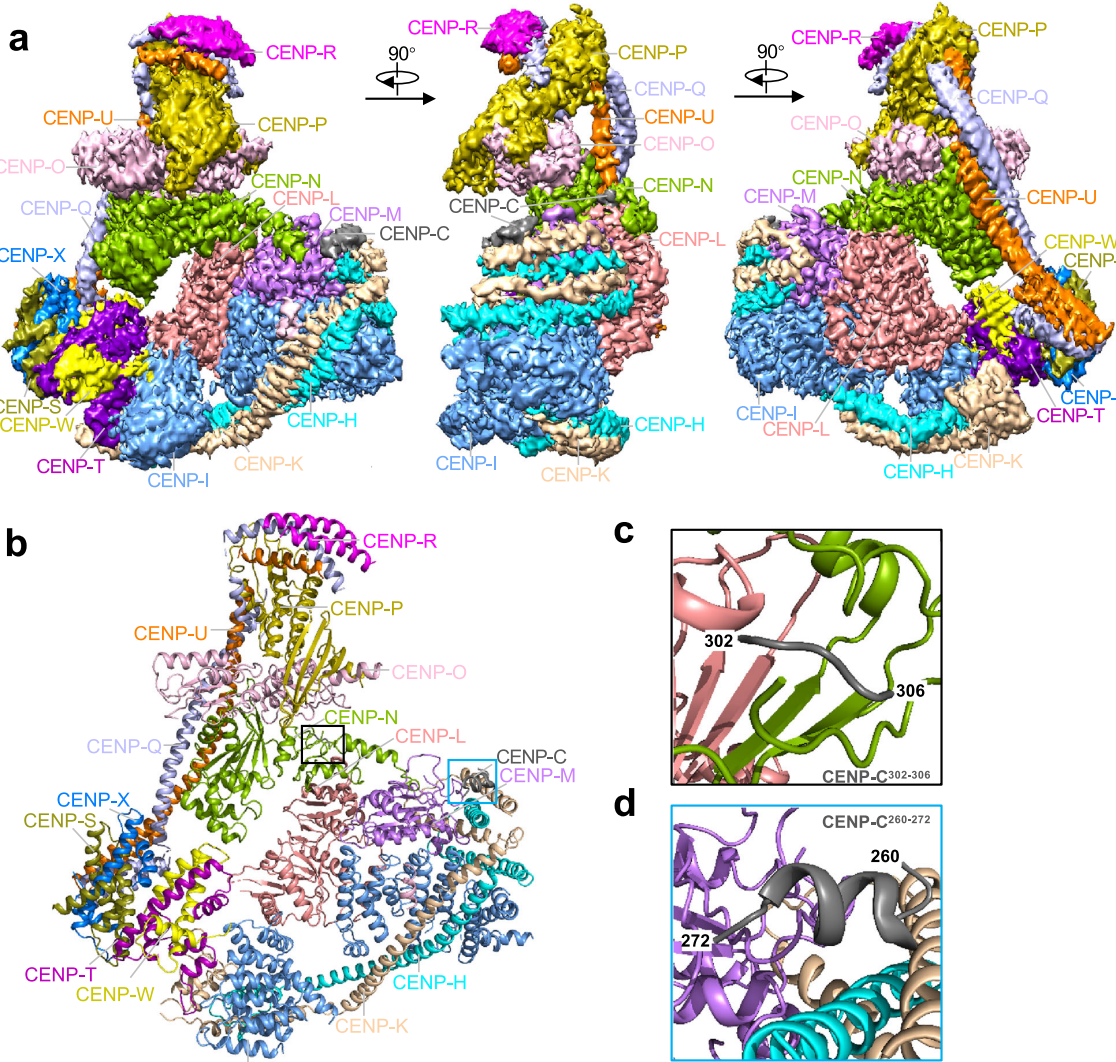
Cryo-EM structure of the human CCAN complex. **a** The 3.3 Å-resolution cryo-EM density map of the CCAN complex at three different views. **b** Cartoon representation of the CCAN structure model. All subunits are assigned into five sub-complexes, including CENP-C, CENP-LN, CENP-HIKM, CENP-OPQUR and CENP-TWSX. The black and blue box showed the fragment of CENP-C. **c**, **d** Enlarged view of the fragment of CENP-C show in **b**. Interface underlying CENP-C^302–306^ interactions with CENP-L (**c**, black frame). CENP-C^260–272^ interacts with CENP-HKM (**d**, blue frame). See also Supplementary Figs. S1–S4.

In the structure of human CCAN, there are four sub-complexes, including CENP-LN, CENP-HIKM, CENP-TWSX, and CENP-OPQUR. The arrangement of the four sub-complexes generates a b-shaped structure, in which CENP-OPQUR adopts an elongated shape to generate the arm, and CENP-LN, CENP-HIKM, and CENP-TWSX form the semi-circle (Fig. 1a). The CENP-LN sub-complex, located at the center of the ‘b’, functions as a node for coordination of the assembly of CCAN by contributing the contact sites, with the sub-complexes CENP-HIKM and CENP-OPQU on the opposite side (Fig. 1b).

The CENP-HIKM sub-complex is comprised of the HIK^head^ and HIK^base^ domains connected by the anti-parallel coiled-coil α-helices from CENP-H and CENP-K to form a V-shaped structure (Fig. 1b; Supplementary Fig. S4a, b). CENP-I folds into two separate domains, both containing five HEAT-repeat like motifs, which associate with helices of CENP-H and CENP-K to form HIK^head^ and HIK^base^ domains, respectively (Supplementary Fig. S4a, b). The architect of human CENP-HIK is similar to that of yeast homolog^21,22^. CENP-M, which adopts a small GTPase-like fold, is located at a pocket on the surface of the HIK^base^ domain formed by the C-terminus of CENP-I, the N-termini of CENP-H and CENP-K, and the CENP-LN sub-complex (Fig. 1b; Supplementary Figs. S3a–d, 4a). Of note, this interaction of CENP-M with CENP-I is different from the previous observation, in which CENP-M is wrapped by CENP-I based on the low-resolution structure determined by negative staining EM^23^. Thus, CENP-M is in a position different from that proposed.

The histone-fold domains of CENP-TWSX form a heterotetramer that shares a similar architecture with the histone H3-H4 tetramer^14,24,25^, within which each subunit contains a histone-fold domain, and which is resolved in our cryo-EM density map (Fig. 1a, b; Supplementary Fig. S3e–h). Despite projection of CENP-TWSX close to CENP-LN and CENP-OPQUR in our cryo-EM structure, biochemical characteristics show that CENP-TWSX interacts only with CENP-HIKM to form an intact submodule (Fig. 1b; Supplementary Figs. S1h–m, S4b). What identified on the surface was a large, positively charged region with the capacity to interact with DNA. It is likely that, in chicken, CENP-TWSX is wrapped by DNA to form a nucleosome-like structure^25^ (Supplementary Fig. S4c, d).

At the CENP-QU C-terminal, parallel α-helix bundles, extending in a hetero-dimeric coiled-coil, form the vertical line of “b” with CENP-OP and CENP-R at the top (Fig. 1a, b). Unlike yeast Nkp1/2^14^, CENP-R does not form a four-helix bundle structure with CENP-QU (Supplementary Fig. S5). Our structural analyses indicate that human CENP-R is not a structural or functional homolog of Nkp1/2 in yeast. Thus, the differences between human and yeast CCAN complexes are minor and perhaps relate to positions and functions of species-specific subunits.

### CENP-C guides the assembly of CCAN complex by tethering CENP-LN and CENP-HIKM

Although we used a fragment of CENP-C containing amino acid residues 180–545 for reconstitution of CCAN on CENP-A nucleosome, most of this fragment of CENP-C is invisible in the cryo-EM map because of its intrinsic disorder. After models of the other 15 CCAN components were built, we found two volumes of unassigned map density close to CENP-LN and CENP-HIKM subcomplexes, respectively (Supplementary Fig. S3i, j; Fig. 1c, d). Assisted by AF2, residues 302–306 of CENP-C were assigned to the density associated with CENP-LN subcomplex in concordance with a previously identified CENP-LN binding motif, which supported the kinetochore recruitment of CENP-LN by CENP-C in vivo (Fig. 1c; Supplementary Fig. S3i)^26^. The fragment includes residues 260–272 of CENP-C, which fit into the density proximal to CENP-HIKM subcomplex (Fig. 1d; Supplementary Fig. S3j). The interaction of CENP-C with CENP-HIKM supported previous observation that mutation of residues 265–267 in CENP-C abolishes the recruitment of CENP-HIKM to kinetochore^27^. Although, in our CCAN complex structure, most of CENP-C is not clearly visible, we observed the interactions of CENP-C with CENP-LN and CENP-HIKM.

### CENP-LN binds to DNA in the CCAN complex

**Fig. 2:**
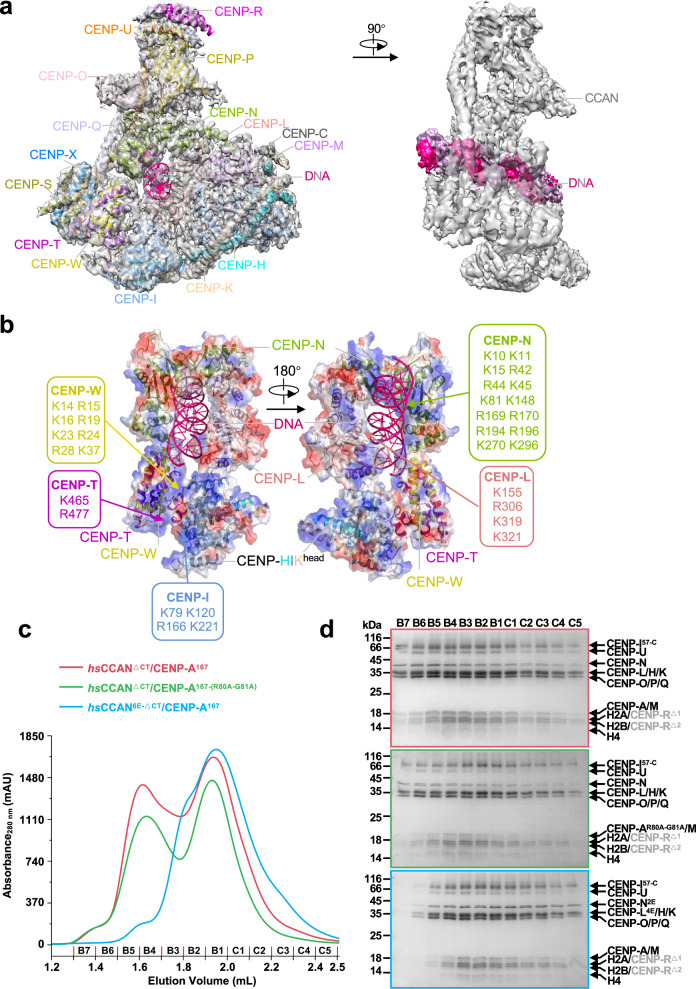
DNA binds to CCAN through the CENP-LN channel. **a** The 3.7 Å resolution cryo-EM density map of CCAN–DNA complex at two different views. The density map of DNA is colored by hotpink and the others are shown in gray transparent surfaces. **b** Electrostatic potential surface view of CENP-LN-HIK^head^-TW binding with DNA. The DNA is shown as cartoon. Note that positively charged amino acids from CENP-LN, CENP-I and CENP-TW constitute the contact sites between CCAN and DNA. **c**, **d** Comparison of elution profiles (**c**) of CCAN^ΔCT^-CENP-A^167^/CCAN^ΔCT^-CENP-A^167-(R80A-G81A)^ and CCAN^6E-ΔCT^-CENP-A^167^ in Superose 6 5/150 GL (GE Health) and the Coomassie-blue stained 15% SDS-PAGE gel. CENP-A^167^ is the CENP-A nucleosome reconstituted by using a DNA fragment of 167 bp in length. **d** The CCAN^ΔCT^ complex bound to either CENP-A nucleosome or CENP-A^R80A-G81A^ nucleosome which reconstituted with 167 bp DNA, but the CCAN^6E-ΔCT^ complex failed. The CCAN^ΔCT^ complex includes CENP-LN, CENP-HIKM and CENP-OPQUR, but not CENP-C and CENP-TWSX; the CCAN^6E-ΔCT^ complex includes charge mutations of positively-charged residues on the CENP-LN (K270E/K296E in CENP-N^2E^, K155E/R306E/K319E/K321E in CENP-L^4E^) in contact with DNA; two degradation products of CENP-R annotated as CENP-R^Δ1^ and CENP-R^Δ2^ in gray color. Of note, the two separated peaks seen in the elution represent wild type CCAN (red line) and CENP-A^Nuc^–binding deficient CCAN (green line) complex with CENP-A^Nuc^ and CENP-A^Nuc^ which are indistinguishable. However, DNA binding-deficient CCAN (cyan line) failed to bind nucleosomal DNA, validating that CCAN binds to DNA via CENP-LN. See also Supplementary Figs. S5, S6.

The structure of CCAN–DNA complex was built into the cryo-EM map at 3.7 Å resolution in which a DNA double helix about 25 bp in length is clearly resolved (Fig. 2a). Around the DNA is a positively charged channel composed by CENP-LN, CENP-HIK^head^, CENP-TW, which is complement to the negative charge of DNA gyre (Fig. 2b). CCAN complex binds to double-strand DNA through the electrostatic interaction between a set of positively charged residues from several CCAN components and the negatively charged phosphate backbone of DNA, which supports the previous reports that functional kinetochore assembly is independent of DNA sequence^10^.

Compared to the structure of *S. cerevisiae* CCAN-CENP-A^Nuc^ complex, both human and yeast CCAN complex bind DNA at the same positively charged grooves assembled by the coordination of CENP-LN sub-complex (Supplementary Fig. S5a, b). However, human CENP-LN has a narrower opening, probably caused by the shorter CENP-N^Cter^ which leads to the formation of a more compact conformation of the human CCAN complex further strengthened by the interaction with CENP-M (Supplementary Fig. S5b–e). Therefore, human CCAN adopts a close ‘b’ shaped conformation to grip double strand DNA, while yeast CCAN forms a more open structure to accommodate CENP-A^Nuc^.

Early studies solved the cryo-EM structures of CENP-LN fragment bound to CENP-A nucleosomes via the RG-loop^26,28,29^. The observed CCAN binding to DNA prompted us to examine whether full-length CENP-N does not bind to the RG-loop of the CENP-A nucleosome in the CCAN complex. We engineered RG-loop mutant CENP-A^R80A-G81A^ and, by use of size-exclusion chromatography (SEC), assessed its impact on nucleosome integrity. As shown in Supplementary Fig. S6a, b, the RG mutant did not interfere with assembly of the CENP-A nucleosome judged by their elution profiles. We next engineered the DNA binding-deficient mutant CENP-LN by reversing the charges on the DNA-binding sites (K155E, R306E, K319E and K321E of CENP-L^4E^; K270E and K296E of CENP-N^2E^) followed by SEC. As shown in Supplementary Fig. S6c, d, the CENP-L^4E^N^2E^ mutant did not interfere with assembly of CCAN judged by its elution profile which is similar to that of CCAN complex with wild-type CENP-LN. We further assessed CCAN binding to DNA using electrophoretic mobility shift assay (EMSA) and confirmed that the CENP-L^4E^N^2E^ mutant was insufficient for mobilizing free DNA compared to wild-type CCAN (Supplementary Fig. S6e, f). By use of SEC, we assessed whether CENP-N binds to the RG-loop mutant CENP-A^R80A-G81A^ nucleosome in the CCAN context. Because CENP-C and CENP-TWSX interact with the C-terminal tail of CENP-A and DNA on CENP-A nucleosome in vitro, respectively^24,30^, we reconstituted the CCAN^ΔCT^ complex (CCAN complex without CENP-C and CENP-TWSX) to assess the binding efficiency of CENP-LN to DNA. As shown in Fig. 2c, d, the elution profile of the CCAN^ΔCT^ complex with the RG-loop mutant CENP-A^R80A-G81A^ nucleosome was indistinguishable from that for the wild-type CENP-A nucleosome, indicating that CENP-N binds poorly to the RG-loop of the CENP-A nucleosome in the CCAN context. However, the elution profile of DNA binding-deficient CCAN^ΔCT^ (CENP-L^4E^N^2E^, called CCAN^6E-ΔCT^ for short) was distinctly different from that of wild-type CCAN^ΔCT^ (Fig. 2c, d), suggesting that the recombinant CCAN complex possesses strong DNA-binding activity in vitro. Thus, we conclude that CENP-N binds to DNA in the CCAN^ΔCT^ complex but less efficient to the RG-loop of CENP-A.

### The binding of CENP-N to DNA determines its centromere localization and function

**Fig. 3:**
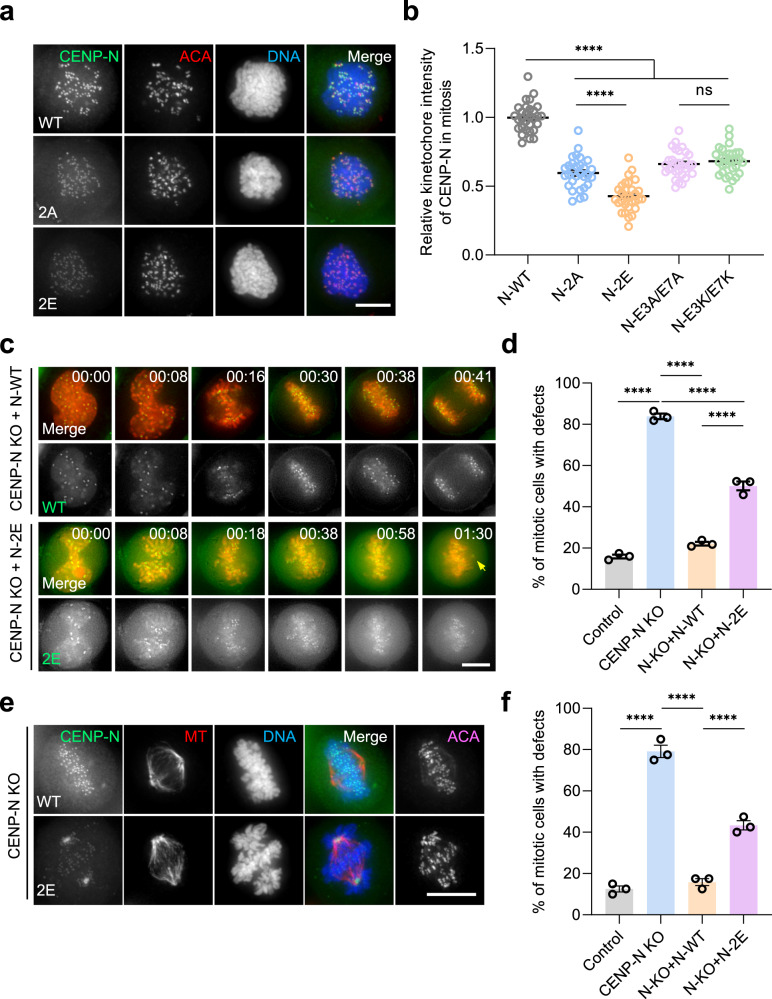
DNA binding is required for CENP-N centromere localization and function in mitosis. **a** Representative immunofluorescence montage of HeLa cells expressing GFP-CENP-N wild type and DNA binding-deficient mutants. Scale bar, 10 µm. Note that K270 and K296 binding to DNA determines CENP-N localization to centromere in mitosis. **b** Statistical analyses of centromere localization efficacy of CENP-N wild type and mutants. Data present means ± s.e.m. from three independent experiments of 30 cells for each group. Ordinary one-way ANOVA followed by Tukey’s post hoc test was used to determine statistical significance. ****p < 0.0001; ns, not significant. **c** Real-time imaging of HeLa cells with chromosome marked by H2B-mCherry and GFP-tagged CENP-N wild type and 2E mutant in the absence of endogenous CENP-N. Note that 2E mutant caused mitotic arrest with chromosome alignment defect. Scale bar, 10 µm. **d** Quantification of mitotic phenotypes in cells expressing CENP-N 2E mutant after induction of endogenous CENP-N knockout as in **c**. Data present means ± s.e.m. from three independent experiments (Control, *n* = 69; CENP-N KO, *n* = 68; N-KO + N-WT, *n* = 68; N-KO + N-2E, *n* = 68). Ordinary one-way ANOVA followed by Tukey’s post hoc test was used to determine statistical significance. ****p < 0.0001. **e** Representative immunofluorescence montage of HeLa cells expressing GFP-CENP-N wild type and 2E mutant and stained for kinetochore microtubule. Scale bar, 10 µm. Note that 2E mutant failed to localize to centromere which resulted in aberrant spindle and misaligned chromosomes. **f** Statistical analyses of chromosome alignment efficacy of CENP-N wild type and 2E mutant. Data present means ± s.e.m. from three independent experiments of 120 cells for each group. Ordinary one-way ANOVA followed by Tukey’s post hoc test was used to determine statistical significance. ****p < 0.0001. See also Supplementary Fig. S7.

Having demonstrated that CENP-N binds to DNA in the human CCAN complex, we next assessed whether DNA binding activity is required for CENP-LN localization to centromeres in HeLa cells. Since CENP-N in CCAN complex binds to DNA but not to the RG-loop of CENP-A judged by SEC, we first assessed if the DNA binding-deficient CENP-N mutants CENP-N^2A^ and CENP-N^2E^ localized to centromere. As shown in Supplementary Fig. S7a, Western blotting analyses showed that various CENP-N variants are expressed at comparable levels in HeLa cells. We next examined the localization of DNA binding-deficient mutant CENP-N^2A^ and CENP-N^2E^ in interphase centromere, as judged by their co-localization to the centromere marker ACA using immunofluorescence microscopic analyses. Although CENP-N^2A^ (K270A/K296A) and CENP-N^2E^ (K270E/K296E) are apparently located to interphase centromere (Supplementary Fig. S7b, c), their localization to the centromere of nocodazole synchronized cells was apparently diminished (Fig. 3a), suggesting that CENP-N localization to centromere in mitosis depends on its DNA binding activity. We next generated RG-loop binding-deficient CENP-N mutants to replace two negatively charged amino acids with neutral or positively charged amino acids (E3/E7, CENP-N^E3K/E7K^ and CENP-N^E3A/E7A^) as previously reported^28^. Consistent with previous observation, localization of CENP-N^E3K/E7K^ and CENP-N^E3A/E7A^ mutants to the centromere of interphase cells was significantly reduced (Supplementary Fig. S7c, d). In addition, the localization of CENP-N^E3K/E7K^ and CENP-N^E3A/E7A^ mutants to the centromere of nocodazole-treated cells was also reduced but not abolished (Fig. 3b; Supplementary Fig. S7e), suggesting that RG-loop binding activity is required but not sufficient for stable centromere localization of CENP-N in mitosis. Immunoprecipitation analyses confirmed that CENP-A binding to CENP-N^E3K/E7K^ was also reduced (Supplementary Fig. S7f), validating that both CENP-N^E3K/E7K^ and CENP-N^E3A/E7A^ mutants exhibited perturbed binding to CENP-A. Our statistical analyses showed that DNA binding is required for stable localization of CENP-N to the centromere of mitotic cells (Fig. 3b).

We next evaluated the functional relevance of CENP-N binding to DNA in real-time chromosome segregation. The knockout efficiency of CENP-N was assessed by western blotting and immunofluorescence using inducible CRISPR/Cas9-mediated CENP-N knockout HeLa cells as reported^31^ (Supplementary Fig. S7g, h). Consistent with the structural analyses and centromere localization efficiency, expression of DNA binding-deficient CENP-N^2E^ in the absence of endogenous CENP-N resulted in chromosome misalignment and mitotic arrest in real-time imaging (Fig. 3c; bottom panel). However, expression of wild-type CENP-N fully rescued aberrant chromosome segregation phenotype seen in endogenous CENP-N-deficient HeLa cells (Fig. 3c; top panel). Statistical analyses showed that wild-type CENP-N minimized the chromosome alignment errors, but CENP-N^2E^ only partially rescued the phenotype (Fig. 3d), suggesting that the DNA-binding activity of CENP-N is critical for faithful chromosome segregation in mitosis. Immunofluorescence microscopic analyses confirm the chromosome segregation defects and aberrant spindle in CENP-N^2E^-expressing cells (Fig. 3e, f). Thus, we conclude that the DNA-binding activity of CENP-N is required for its stable localization to centromere and accurate chromosome segregation.

### The CENP-L binding to DNA is essential for accurate chromosome segregation

**Fig. 4:**
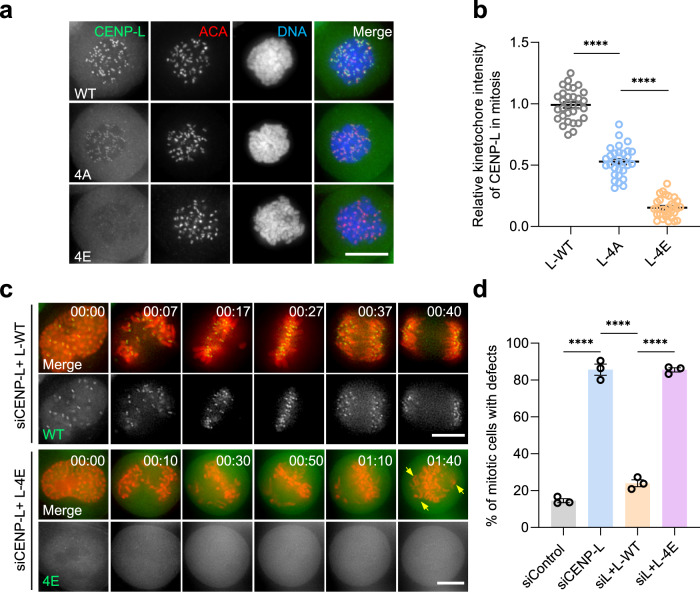
CENP-L binding to DNA is essential for accurate chromosome segregation. **a** Representative immunofluorescence montage of HeLa cells expressing GFP-CENP-L wild type and DNA binding-deficient mutants. Scale bar, 10 µm. Note that K155/R306/K319/K321 determine CENP-L localization to centromere in mitosis. **b** Statistical analyses of centromere localization efficacy of CENP-L wild type and mutants (4A, 4E). Data present means ± s.e.m. from three independent experiments of 30 cells. Ordinary one-way ANOVA followed by Tukey’s post hoc test was used to determine statistical significance. ****p < 0.0001. **c** Real-time imaging of HeLa cells with chromosome marked by H2B-mCherry and GFP-tagged CENP-L wild type and 4E mutant in the absence of endogenous CENP-L. Note that 4E mutant caused mitotic arrest with chromosome alignment defects. Scale bar, 10 µm. **d** Quantification of mitotic phenotypes in cells expressing CENP-L 4E mutant after induction of endogenous CENP-L knockout as in **c**. Data present means ± s.e.m. from three independent experiments of (siControl, *n* = 67; siCENP-L, *n* = 68; siL + L-WT, *n* = 67; siL + L-4E, *n* = 69). Ordinary one-way ANOVA followed by Tukey’s post hoc test was used to determine statistical significance. ****p < 0.0001. See also Supplementary Fig. S8.

Previous studies demonstrate that recombinant CENP-LN heterodimer forms a node at the centromere-kinetochore interface^26,28,29^. However, these studies lack the structural insights of the CENP-N C-terminus due to its folded structure and absence of its interactions with CCAN including CENP-L^26,28,29^. In these studies, however, the molecular basis of human CENP-LN heterodimer organization and its function in CCAN integrity and centromere function were examined. In the CCAN complex, CENP-L interacts with CENP-N to form a U-shaped structure with an opening of about 25 Å, which is less than half that for yeast homolog (Supplementary Fig. S5). Since our cryo-EM analyses of CCAN revealed the CENP-LN structure, we examined whether DNA-binding activity is necessary for CENP-L localization and function in centromere. To this end, we engineered two CENP-L mutants in which four positively charged amino acids (K155, R306, K319 and K321) in DNA contact were replaced with no charges or negatively charged amino acids, called CENP-L^4A^ and CENP-L^4E^, respectively. As shown in Fig. 4a and Supplementary Fig. S8a, b, the centromere localizations of those mutants were reduced due to their DNA-binding deficiency. Statistical analyses showed that both CENP-L^4A^ and CENP-L^4E^ failed to stably localize to the centromere (Fig. 4b). To assess the importance of the CENP-L binding to DNA in mitosis, we examined whether CENP-L wild-type and CENP-L^4E^ could rescue the phenotypes associated with the deficiency of endogenous CENP-L. As shown in Supplementary Fig. S8c, chromosome misalignment in endogenous CENP-L-deficient cells were corrected by wild-type CENP-L but not by DNA binding-deficient mutant CENP-L^4E^. In CENP-L^4E^-expressing cells depleted of endogenous CENP-L, chromosomes failed to align at the spindle equator. Statistical analyses showed that expression of CENP-L^4E^ resulted in chromosome misalignment (Supplementary Fig. S8d). Thus, we conclude that CENP-L binding to DNA is essential for chromosome alignment in mitosis.

We next examined how DNA binding-deficient CENP-L affects chromosome dynamics during mitosis. As shown in Fig. 4c, real-time imaging indicated that wild-type CENP-L rescued chromosome segregation defects in endogenous CENP-L-suppressed cells (top panel). However, chromosome segregation defects were not rescued by DNA binding-deficient mutant CENP-L^4E^ (Fig. 4c; bottom panel, arrows). Statistical analyses showed that CENP-L binding to DNA is essential for accurate chromosome segregation (Fig. 4d). Thus, we conclude that the CENP-LN binding to DNA is essential for centromere localization and function in mitosis.

## Discussion

Our study of the human CCAN–DNA interaction defines the molecular contacts between the fundamental module of the kinetochore and DNA. In both CCAN and CCAN–DNA complex structures, CCAN adopts a ‘b’ shaped structure which is different from the structure of yeast CCAN^14^. In our CCAN–DNA structure, CENP-LN coordinates to form a closed channel with positive charge to encompass a double-strand DNA which is similar to CENP-LN interaction with unwrapped DNA in yeast CCAN-CENP-A^Nuc^. Previously solved structure of N-terminal domain of CENP-N (CENP-N^Nter^) in complex with CENP-A^Nuc^ revealed that CENP-N recognize CENP-A^Nuc^ through the interaction with RG-loop of CENP-A^26,28,29^. Our structural and biochemical analyses show that the RG-loop of CENP-A is required for CCAN loading but not sufficient for CCAN localization to centromere and function in mitosis. Thus, the information from these structures suggests that the interaction of CENP-LN alone with CENP-A^Nuc^ is different from that of CENP-LN in CCAN complex.

Our cryo-EM analyses of the CCAN–DNA complex has revealed structure-functional relationship between the human and yeast CCAN complexes (Supplementary Fig. S5a, b). First, the presence of CENP-M and CENP-R in human CCAN provides a unique landmark for human kinetochores which is structurally distinct from yeast CCAN^32^. Furthermore, CENP-LN adopts a more compact conformation of the human CCAN complex compared to their yeast counterparts. In addition, DNA was bound in the central pore of the closed structure of the human CCAN complex. Although human CENP-TWSX is located adjacent to CENP-OPQUR and CENP-HIKM, it is possible for human CENP-TWSX to form a nucleosome-like structure even in the intact CCAN complex similar to that of chicken CENP-TWSX alone^24,25^.

Our structural analyses suggest that human CCAN might bind DNA only after assembled into an integral protein machine. Given the fact that the assembly of CCAN is a stepwise process^15^, we speculate that free CENP-LN heterodimer is recruited to centromere exclusively through the recognition of CENP-A RG-loop at the early stage of cell cycle. Once other CCAN components are recruited by CENP-LN and CENP-C, the occurrence of a large conformational change of CENP-LN in fully assembled CCAN complex results in the formation of a positively charged loop to accommodate double-stranded DNA helix. Less compact centromeric chromatin caused by dilution of CENP-A in the interphase supports the transition of CENP-N from CENP-A protein to centromeric DNA^33^. During the revision of this study, two related works on cryo-EM analyses of human CCAN were released and those structures agree with our CCAN overall architecture^34,35^. Similarly, a central positively charged channel composed of CENP-LN, CENP-HIK^head^ and CENP-TWSX was identified in the reported structures with the ability to binds DNA. The duplex DNA we visualized was topologically entrapped by human CCAN while Barford and colleagues observed the CCAN complex with extra-nucleosomal linker DNA of CENP-A nucleosome^34^. Interestingly, the centromere localization of N2E and L4E mutants was reduced much more significantly than N2A and L4A, respectively, in our study. Because K270 and K296 of CENP-N and K155, R306, K319 and K321 of CENP-L are positively charged, mutation of these positively charged residues to L4E results in repulsion force and steric hindrance between CCAN and DNA. Although we analyzed three overall similar CCAN structures reconstituted in vitro^34,35^, we do not yet understand the physiological relevance of the DNA-bound CCAN in live cells and how CENP-N switches from CENP-A RG loop-binding to centromeric DNA association during CCAN assembly associated with cell cycle progression. Thus, despite the constitutive presence of CCAN components at the centromere, the cell cycle machinery may guide the centromere remodeling and turnover to adopt a cell cycle stage-dependent function via post-translational modifications^36–38^. More recently, it was reported that CENP-N is involved in nucleosome compaction in vitro^39^. Thus, future studies will fill in the gap between biochemical validation of CCAN structure and cellular delineation of its function and assembly dynamics during cell cycle.

In summary, our analyses of the human CCAN structure-activity relationship revealed a context-dependent CENP-N localization and function at centromere. CENP-N binds to the RG loop of CENP-A in interphase but switches to DNA-based centromere localization upon CCAN assembled in mitosis. The challenge ahead is to use single-molecule analyses to visualize the respective conformational flexibility of the CENP-LN dimer and relate the spatiotemporal dynamics of assembly of CCAN to the mechanism of action underlying kinetochore function in mitosis. Finally, characterization of the interfaces of human CCAN structural elements will advance our understanding of its relevance in various contexts such as aneuploidy and chromothripsis^40,41^.

## Materials and methods

### Cloning and protein expression

Full-length CENP-N, with a 6× His-tag fused to its C-terminus, and CENP-L were cloned into a Multi-Bac vector pACEBac1 (Invitrogen) or a pIDC vector, respectively. Then, two plasmids were fused by a Cre-LoxP reaction; expression of the CENP-LN complex was assessed using a MultiBac^Turbo^ expression system. The 180–545 truncation of CENP-C (CENP-C^180–545^) was cloned into pFastBac1 with a glutathione-S-transferase (GST)-tag in its N-terminus to generate the CENP-LNC^180–545^ complex by co-infection with CENP-LN P3 virus in Sf9 insect cells. The plasmid constructed for CENP-H and -K was similar to that for CENP-LN. For expression of the CENP-HIKM complex, CENP-HK P3 and CENP-HIKM P3 virus were co-infected into Hi5 insect cells as previously reported^15^. To generate the CENP-OPQUR subcomplex, CENP-O, CENP-P, and CENP-Q were cloned into Multi-Bac vector pACEBac1 with a 6× His-tag fused to the C-terminus of CENP-Q, and CENP-U and CENP-R were cloned into the pIDC plasmid, then the two plasmids were fused by the Cre-LoxP reaction. For expression of the CENP-TWSX complex, the genes encoding full-length human CENP-T and CENP-W were first cloned into Multi-Bac vector1 pACEBac1 (Invitrogen) with a 6× His-tag fused to the N-terminus of CENP-T, and then CENP-S and CENP-X were cloned into Multi-Bac vector1 pIDC with a 6× His-tag fused to the N-terminus of CENP-S. Subsequently, the two plasmids were fused by the Cre-LoxP reaction. The baculoviruses for expression of CCAN subcomplexes were prepared for expression using the Bac-to-Bac® Baculovirus Expression System. We used the baculoviral stock to infect Sf9 insect cells for expression of the CENP-LN, CENP-OPQUR, and CENP-TWSX subcomplexes at 27 °C for 48~72 h. To obtain more of the CENP-HIKM subcomplex, we co-infected the P3 baculoviruses of CENP-HIKM and CENP-HK with a 4:1 volume ratio into Hi5 insect cells. All plasmids were sequenced for verification.

To construct pLVX-EGFP-CENP-N or CENP-L plasmid, the CENP-N/L cDNA was amplified by PCR and digested by *EcoR*I and *Bam*HI and cloned into pLVX-EGFP-C1 vector, respectively. CENP-A was cloned by PCR and inserted into p3×FLAG-Myc-CMV-24 expression vector (Sigma). To generate siRNA-resistant CENP-L constructs, two synonymous mutations were generated (R306R/V307V). To generate sgRNA-resistant CENP-N plasmid, the corresponding sgRNA PAM sequence in cDNA was eliminated through a synonymous mutation (V152V, GTG to GTA). All the mutations were generated by Mut Express II Fast Mutagenesis Kit (Vazyme) according to the manufacturer’s instructions. And all plasmids with the desired insertions or mutation were sequenced at General Biosystems.

### Protein purification

Sf9 and Hi5 cells were used for expression (72 h, 27 °C), after which the cells were harvested by centrifugation and suspended in lysis buffer. Cells were lysed with high-pressure in lysis buffer (50 mM HEPES-Na, pH 7.5; 500 mM NaCl; 5 mM imidazole; 10% glycerol, and 2 mM β-mercaptoethanol (ME)) supplemented with 2 μM pepstatin A, 10 μM leupeptin, 1 mM benzamidine, and 1 mM phenylmethylsulfonyl fluoride. All protein samples were purified by nickel-nitriloacetic acid (Ni-NTA, QIAGEN) affinity chromatography. Then CENP-C^180–545^-LN and CENP-LN complexes were purified by size-exclusion chromatography (SEC) using a Superdex 200 (10/300) increase (GE Healthcare) column with SEC buffer (20 mM HEPES-Na, pH 7.5; 300 mM NaCl; 0.5 mM tris(2-carboxyethyl)-phosphine (TCEP)). CENP-OPQUR and CENP-TWSX complexes were purified with a Mono S 5/50 GL column (GE Healthcare) with IEX buffer 1 (50 mM HEPES-Na, pH 7.5; 300 mM NaCl; 1 mM dithiothreitol (DTT); and 50 mM HEPES-Na, pH 7.5; 1 M NaCl; 1 mM DTT), respectively, and further purified by a Superdex 200 (10/300) increase (GE Healthcare) column with SEC buffer. In contrast, purification of the CENP-HIKM complex was accomplished with a Mono Q 5/50 GL column (GE Healthcare) and IEX buffer 2 (50 mM HEPES-Na, pH 7.5; 100 mM NaCl; 1 mM DTT; and 50 mM HEPES-Na, pH 7.5; 1 M NaCl; 1 mM DTT) and further purification by SEC using the same column and SEC buffer with other subcomplexes. Peak fractions of all protein samples purified by SEC were collected and stored in liquid nitrogen for complex assembly.

### Reconstitution of CENP-A nucleosome

Cloning, expression, and purification of CENP-A nucleosomes were as described previously^42^. The genes encoding full-length human H2A-H2B and CENP-A-H4 were cloned into a pET28K and a pETDuet-1 vector, respectively. The two plasmids were co-transformed into *E. coli* Rosetta2 (DE3) cells, which were plated on agar containing 50 μg/mL kanamycin and 100 μg/mL ampicillin and grown overnight at 37 °C. Picked monoclones were inoculated into a 5-mL starter culture of Luria-Bertani (LB) media and grown for 3 h at 37 °C. The starter culture was transferred into 50 mL LB medium and incubated at 37 °C for another 3 h. The second culture (15 mL) was transferred into 1 L of LB medium and incubated at 37 °C to an OD_600_ of ~0.5. The culture was induced with 0.4 mM isopropyl β-D-1-thiogalactopyranoside and incubated at 37 °C for 12–16 h. Cells were harvested and lysed using high pressure in lysis buffer A (20 mM Tris-HCl; 2 M NaCl; 1 mM β-ME, pH 8.0). The histone octamer was first purified by Ni-NTA (QIAGEN) affinity chromatography, and the (His)8-tag was removed by TEV protease overnight at 4 °C. It was further purified using a HiLoad Superdex200 (16/60) (GE Healthcare) column with SEC buffer (20 mM Tris, 2 M NaCl, 1 mM DTT, pH 8.0), and the peaks corresponding to histone octamers were assessed by SDS-PAGE. Purified histone octamers were wrapped with a 147-bp ‘Widom 601’ DNA fragment to reconstitute the CENP-A nucleosome by dialysis and used for further studies^43^. The sequence of the 147-bp ‘Widom 601’ DNA fragment is shown below:

ATCGAGAATCCCGGTGCCGAGGCCGCTCAATTGGTCGTAGACAGCTCTAGCACCGCTTAAACGCACGTACGCGCTGTCCCCCGCGTTTTAACCGCCAAGGGGATTACTCCCTAGTCTCCAGGCACGTGTCACATATATACATCCGAT.

In some instances, 167-bp DNA was used for in vitro reconstitution experiments. The reconstitution of CENP-A nucleosome with a 167-bp DNA (CENP-A^167^) was the same as mentioned above. The sequence of the 167-bp DNA fragment is listed below:

ACTTACATGCACAGGATGTATATATCTGACACGTGCCTGGAGACTAGGGAGTAATCCCCTTGGCGGTTAAAACGCGGGGGACAGCGCGTACGTGCGTTTAAGCGGTGCTAGAGCTGTCTACGACCAATTGAGCGGCCTCGGCACCGGGATTCTCCAGGGCGGCCAGT.

### Preparation of the human CCAN-CENP-A^Nuc^ complex

The CENP-LNC^180–545^ complex was first mixed with CENP-A nucleosome with a molar ratio of 1.5:1 in 20 mM HEPES-Na, pH 7.5; 300 mM NaCl; and 0.5 mM TCEP. The CENP-HIKM, CENP-OPQUR, and CENP-TWSX complexes were then added with the same molar ratio (CENP-A^Nuc^: CENP-LNC^180–545^: CENP-HIKM: CENP-OPQUR: CENP-TWSX = 1.5:1:1:1:1) for 1 h at 4 °C. The assembled complex was then purified using a Superose 6 (10/300) (GE Healthcare) column equilibrated with SEC buffer (20 mM HEPES-Na, pH 7.5; 300 mM NaCl; 0.5 mM TCEP). The purified CCAN-CENP-A^Nuc^ complex was cross-linked with 5 mM bissulfosuccinimidyl suberate (BS3, Thermo Fisher Scientific) for 1 h on ice, quenched with 50 mM Tris, and further subjected to SEC with a Superose 6 (10/300) (GE Healthcare) column for cryo-EM analysis. A CENP-LN subcomplex was used for assembly of a CCAN (LN)-CENP-A^Nuc^ complex in the same way as the CCAN-CENP-A^Nuc^ complex. The CCAN complex was reconstituted by mixing purified CENP-LNC, CENP-HIKM, CENP-OPQUR, and CENP-TWSX; the sample preparation was the same as for the CCAN-CENP-A^Nuc^ complex.

### Cryo-EM data collection and processing

The CCAN-CENP-A^Nuc^ complex was applied to a glow-discharged holey carbon grid (Quantifoil, R1.2/1.3, 300 mesh) in an aliquot of 3 μL of fresh sample at a concentration of 1 mg/mL (measured by Nanodrop at 260 nm). The grids were blotted for 5 s; maintained for 40 sec under 100% humidity at 4 °C; and plunged into liquid ethane using an FEI Vitrobot (FEI Company).

The Titan Krios G3i cryo electron microscopes operating at 300 kV equipped with a Gatan K2 Summit camera, were used for data collection. The cryo-EM data were collected by *SerialEM* with the under-focus range of 1.7~2.7 μm and at magnification of SA22, 500× in super-resolution mode, corresponding to a pixel size of 0.61 Å on the sample level. The dose rate was set to be ~10 electrons per physical pixel per second and the total exposure time for each movie was 7.68 s, fractioned to 32 frames, resulting in a total dose of 50 electrons/Å^2^.

The image processing workflow is illustrated in Supplementary Fig. S2f. In brief, 12,643 movie stacks were motion-corrected and dose-weighted by MotionCor2^44^. Defocus values were estimated by CTFFIND4^45^. After manually checking, good micrographs were selected for automatically particle picking with references generated from published 3D volume of yeast CCAN complex using RELION3^46^. Picked particles were extracted and applied to non-supervised 2D classifications. Selected particles were then imported into cryoSPARC (v.3.2.0) for subsequent image processing^47^. After ab initio 3D classification into 3 classes, 39,863 particles were selected for non-uniform refinement, which gave an EM map at 4.5 Å. Particles were subjected to 2D classification and good classes were selected as template for automatic particle picking. 2,519,574 particles were auto-picked and subjected to several rounds of 2D classifications to exclude junk particles and contaminations. In total, 1,370,060 particles were selected for 3D reconstruction. Ab initio reconstruction was accomplished with five designated classes. The best class displayed clear features of the complex, and the selected particles were submitted to non-uniform refinement. Finally, a map with an overall resolution of 3.3 Å was achieved. Further ab initio reconstructions into two classes resulted in two maps with various DNA occupancies. The one without DNA density in the binding pocket was refined to 3.5 Å, and the one with DNA was refined to 3.7 Å. All reported resolutions were calculated on the basis of a 0.143 Fourier shell correlation cut-off following gold-standard refinement^48^. Local resolution variations were estimated using ResMap^49^.

### Model building and structure refinement

For model building of a CCAN complex without DNA, the Cryo-EM density maps were visualized in Chimera and Coot^50,51^. We combined de novo modeling, homology modeling, and rigid-body fitting of subunits with known structures to generate an atomic model. An initial crude model was auto-built combining the results of software Buccaneer in CCPEM program suit^52^ with that of *phenix.trace_and_build* command in the Phenix package^53,54^ against the cryo-EM map. Bundles of helices were built automatically. The atomic model of the C terminus of CENP-N, full-length CENP-L, CENP-H (residues 39–241), CENP-I (residues 62–654), and CENP-K (residues 20–268) was built *de novo* combining the information of these auto-built helices, and secondary-structure prediction information from PSIPred analysis^55^. Sequence identification and assignments of these subunits were guided mainly by aromatic or bulky residues such as His, Tyr, Phe, and Trp. The crystal structure of the N-terminal domain of human CENP-N (PDB:6EQT, residues1–211)^26^ and the human CENP-M (PDB: 4P0T, residues 1–170)^23^ were directly docked into the cryo-EM map by rigid-body fitting using UCSF Chimera. Then they were refitted and refined according to the good density in Coot. The structure of CENP-O (residues 112–300), which was generated from the full-length CENP-O structure predicted by I-TASSER^20^, was docked into the cryo-EM map by rigid-body fitting using UCSF Chimera. Then they were refitted and refined according to the good density in Coot. By contrast, CENP-P (residues 76–288) performed the preceding operations as CENP-O. The crystal structure of the chicken CENP-TWSX hetero-tetramer (PDB: 3VH5)^25^, corresponding to residues CENP-T (458–556), CENP-W (14–88), CENP-S (10–106), and CENP-X (8–81), were overall fitted into the cryo-EM density map, then refitted, built, manually adjusted and mutant to human species sequence according to the density in Coot. For CENP-Q and CENP-U, it was also de novo built while assigned as alanine due to the lack of adequate information of side chain to identify the correct sequence. Maps without post-processing were used to build the unassigned chain. The DNA duplex of the CCAN–DNA complex was created by fitting a 20-bp long DNA from the unwrapped nucleosomal DNA in the *S. cerevisiae* CCAN-CENP-A^Nuc^ complex (PDB: 6QLD)^14^ into the cryo-EM density map at 3.7 Å resolution.

The structure model of the overall-CCAN complex and CCAN–DNA complex are refined against the 3.3 Å and 3.7 Å overall map in real space with PHENIX and validated by MolProbity^56^. The statistics of the map reconstruction and model refinement are summarized in Supplementary Table S1. All figures were prepared using Chimera^50^ and Pymol (Molecular Graphics System, v2.5, Schrödinger).

### Analytical SEC of CCAN/CENP-A^Nuc^ complex and mutants

CENP-LN or CENP-L^2E^N^4E^, CENP-HIKM and CENP-OPQUR complex were first mixed with a molar ratio of 1:1:1 to form the CCAN^ΔCT^ or CCAN^6E-ΔCT^ complex. The CCAN^ΔCT^ was then mixed with either CENP-A^167^ or CENP-A^167-(R80A-G81A)^ nucleosome with a molar ratio of 2:1 and the CCAN^6E-ΔCT^ was mixed with CENP-A^167^ nucleosome with the same molar ratio for one hour at 4 °C. Different assembled complexes were then performed using a Superose 6 Increase 5/150 GL (GE Healthcare) column equilibrated with SEC buffer (20 mM HEPES-Na, pH 7.5, 300 mM NaCl, 0.5 mM TCEP). Elution of proteins was monitored at 280 nm. Fractions (100 μL/tube) were collected and analyzed by 15% SDS-PAGE gel, then stained with Coomassie Brilliant Blue.

### EMSA

The purified CCAN^ΔCT^ and CCAN^6E-ΔCT^ complexes were respectively incubated with 147 bp DNA in SEC buffer (20 mM HEPES-Na, pH 7.5, 300 mM NaCl, 0.5 mM TCEP) for 10 min on ice. After adding 6% sucrose in the mixtures, samples were analyzed by 1% agarose (w/v) at 120 V for 25 min in 0.5× TAE buffer. The gels were stained with GelRed and visualized using Tanon-1600 imaging system (Tanon Science & Technology) at UV mode.

### Cell culture, synchronization, and stable cell line generation

HeLa and HEK293T cells were maintained in DMEM (Gibco) with 10% fetal bovine serum (FBS, Hyclone) and 100 units/mL penicillin (Gibco) plus 100 μg/mL streptomycin (Gibco). The inducible CRISPR/Cas9-mediated CENP-N knockout HeLa cells from Dr. Iain Cheeseman’s laboratory (MIT) were maintained in DMEM (Gibco) with 10% FBS (Tet-tested, Atlanta Biologicals) and 100 units/mL penicillin (Gibco) plus 100 μg/mL streptomycin (Gibco) supplemented with 50 μg/mL G418 (Sigma) and 2.5 μg/mL puromycin (Sigma). Doxycycline (1 μg/mL, Sigma) was used to induce CENP-N knockout^31^. Thymidine-synchronized cells were released for 7 h before being placed onto temperature-controlled chamber for real-time imaging as previously reported^57,58^.

To generate lentivirus expressing GFP-CENP-N/L, pLVX-EGFP-CENP-N/L (WT and different mutants) was cotransfected into HEK293T cells together with pMD2.G and psPAX2 plasmids. Forty-eight hours after transfection, the supernatant was collected and used to infect HeLa cells. HeLa cells stably expressing GFP-CENP-N/L were selected and maintained in DMEM containing puromycin (2.5 μg/mL).

For the purpose of CENP-N WT and 2E mutant rescue experiment, the inducible CRISPR/Cas9-mediated CENP-N knockout HeLa cells were infected with lentivirus expressing sgRNA-resistant GFP-CENP-N-WT/2E and the GFP-CENP-N-WT/2E expressing stable cell lines were isolated by single-cell sorting (BD LSRFortessa). Three days after doxycycline treatment, cells were transfected with mCherry-H2B expressing plasmid. Twenty-four hours later, cells were treated with MG132 for 2 h, then cells were fixed and stained to examine the chromosomal alignment. In case of live cell imaging analysis, cells were cultured in MetTek glassed bottom culture dishes. Before imaging, medium was changed to CO_2_-independent medium (Gibco).

Transfection of plasmids and siRNA into cells was performed with Lipofectamine RNAiMAX (Invitrogen) according to the user’s manual. The siRNA against CENP-L (AAGAUUAGUUCGUGUUUCA) was obtained from GenePharma and was previously confirmed^59^. To analyze the phenotype of CENP-L WT/4E, GFP-CENP-L WT/4E stable cells were transfected twice with siRNA against CENP-L for 60 h. For live cell imaging, 36 h after siRNA transfection, second siRNA treatment was co-transfected with mCherry-H2B plasmid.

### Drug treatment

Thymidine (T9250, 2 mM), Nocodazole (M1404, 100 ng/mL) and MG132 (C2211, 20 μM) were from Sigma. Puromycin was from Thermo (A1113802).

### Antibodies

Anti-α-tubulin (mouse, DM1A, Sigma 05-829) and ACA (anti-centromere antibody, Immunovision HCT-0100) were used for immunofluorescence. Anti-CENP-N antibody were kindly gifted by Dr. Iain Cheeseman. Antibodies used for Western blots were anti-α-tubulin (mouse, DM1A, Sigma 05-829, 1:5,000), anti-β-Actin (Servicebio, GB12001), anti-FLAG-tag (Sigma F1804, 1:2,000), anti-GFP (Proteintech, 50430-2-AP, 1:1,000). The appropriate secondary antibodies were purchased from Jackson ImmunoResearch Laboratories and used as instructed by the vendor’s instruction.

### Immunoprecipitation

For FLAG-tagged protein immunoprecipitation, transfected cells were collected and lysed in lysis buffer (20 mM HEPES, pH 7.4; 150 mM NaCl; 1 mM EDTA; 0.1% Triton X-100) supplemented with protease inhibitor cocktail (Sigma). Cell lysates were clarified by centrifugation and incubated with FLAG-M2 resin (Sigma) at 4 °C with gentle rotation. After washing with lysis buffer three times, the FLAG beads were boiled and assessed by western blotting as previously reported.

### Immunofluorescence microscopy, image processing, and quantification

HeLa cells grown on coverslips were fixed and permeabilized simultaneously with PTEMF buffer (50 mM Pipes (pH 6.8), 0.2% Triton X-100, 10 mM EGTA, 1 mM MgCl_2_, 4% formaldehyde) at room temperature and were processed for indirect immunofluorescence microscopy. Samples were examined on a DeltaVision microscope (Applied Precision) with a 60× objective lens, NA = 1.42, with optical sections acquired 0.25 μm apart in the *z* axis. Deconvoluted images from each focal plane were projected into a single picture using Softworx (Applied Precision). Images were taken at identical exposure times within each experiment and were acquired as 16-bit gray-scale images. After deconvolution, the images were exported as 24-bit RGB images and processed in Adobe Photoshop. Images shown in the same panel have been identically scaled. Kinetochore intensities were measured in ImageJ (rsb.info.nih.gov/ij/) on nondeconvoluted images. The levels of kinetochore-associated proteins were quantified as described previously^60^. In brief, the average pixel intensities from at least 100 kinetochore pairs from five cells were measured, and background pixel intensities were subtracted. The pixel intensities at each kinetochore pair then were normalized against ACA pixel values to account for any variations in staining or image acquisition. Unless otherwise specified, the values for treated cells then were plotted as a percentage of the values obtained from cells of the control groups.

### Time-lapse imaging

For time-lapse imaging, HeLa cells were cultured in glass-bottom culture dishes (MatTek) and maintained in CO_2_-independent medium (Gibco) supplemented with 10% FBS and 2 mM glutamine. During imaging, the dishes were placed in a sealed chamber at 37 °C. Images of living cells were taken with DeltaVision microscopy system. For presentation of details of the real-time imaging, projection images were constructed from a 0.5-µm/section for 3 sections within a cell.

### Statistics and reproducibility

All experiments were performed and repeated independently with similar results for three times. Statistical analyses were performed with GraphPad Prism 8. All statistics were described in the figure legends. No statistical method was used to predetermine sample size. Images were mounted in figures using Photoshop (Adobe).

## Supplementary information

Supplementary Figure S1-8 and Table S1

## Data Availability

The authors declared that all data supporting our findings in this study were available in this paper and Supporting Information.
