# Supplementary material for: Structural insights into human CCAN complex assembled onto DNA
Source: Cell Discov. 2022 Sep 9;8:90. doi: 10.1038/s41421-022-00439-6 (PMC9463443; doi:10.1038/s41421-022-00439-6)
Supplement: Supplementary file 1 — Supplementary Figure S1-8 and Table S1 [file 41421_2022_439_MOESM1_ESM.pdf]

Supplementary Figure 1

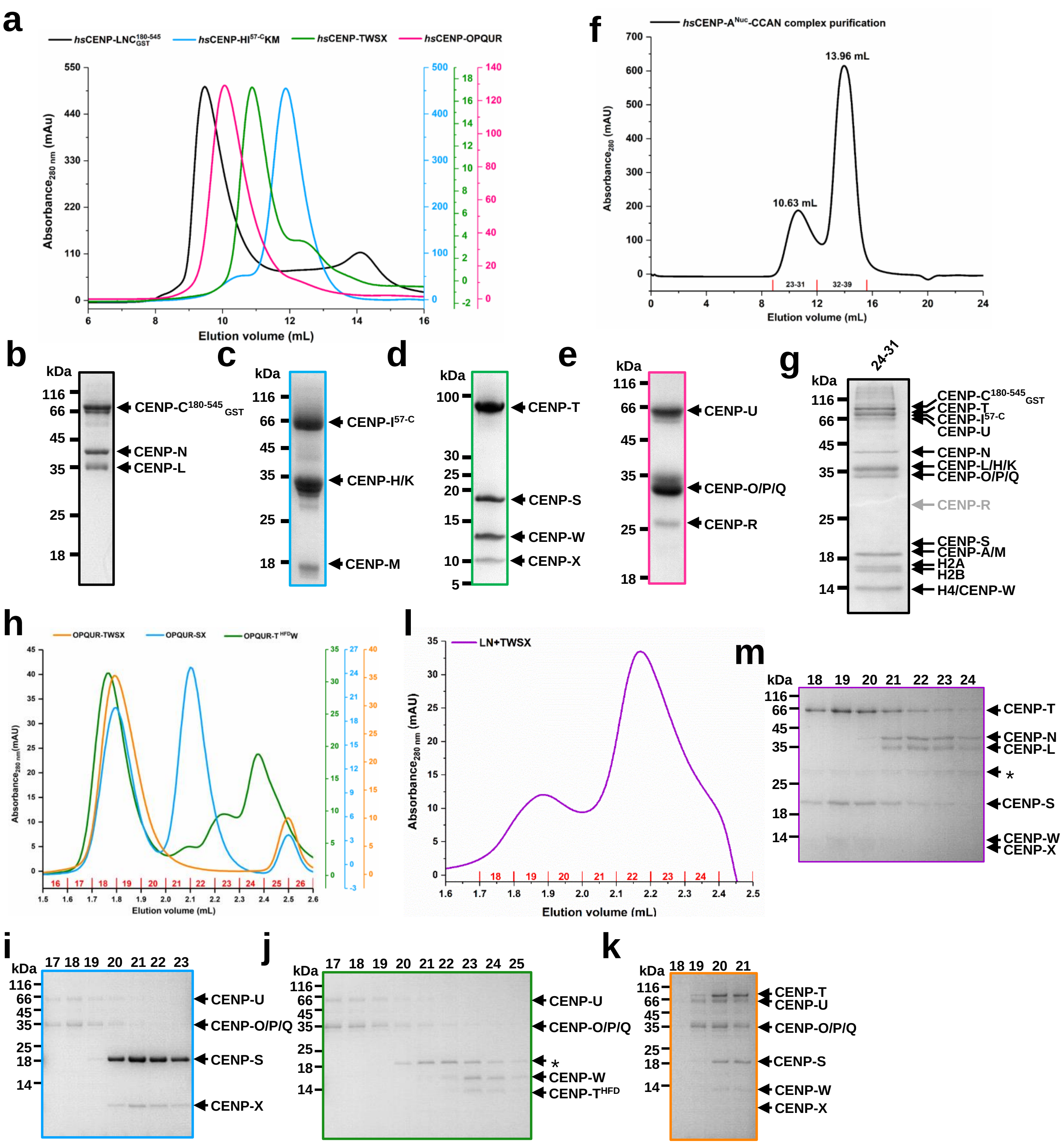

Supplementary Figure 1| Reconstitution of human CCAN-CENP-A^{Nuc} complex *in vitro*.

(**a-e**) Size-exclusion chromatogram profiles for four sub-complexes separated in Superdex 200 10/300 GL (**a**, GE health) and the Coomassie blue stained 15% SDS–PAGE gel of CENP-LNC (**b**), CENP-HIKM (**c**), CENP-TWSX (**d**) and CENP-OPQUR (**e**). All four sub-complexes exhibit distinct elution volumes.

(**f-g**) Size-exclusion chromatogram profiles for CCAN/CENP-A^{Nuc} purified in Superdex 200 10/300 GL (**f**) and the Coomassie blue stained 15% SDS–PAGE gel (**g**).

(**h-k**) CENP-TWSX sub-complex does not bind to CENP-OPQUR. Size-exclusion chromatogram profiles for CENP-TWSX(or CENP-T^{HFD}W or CENP-SX)/CENP-OPQUR (**h**) complex and the Coomassie blue stained 15% SDS–PAGE gel (**i-k**).

(**l-m**) CENP-TWSX sub-complex does not bind to CENP-LN. Size-exclusion chromatogram profiles for CENP-TWSX/CENP-LN complex (**l**) and the Coomassie blue stained 15% SDS-PAGE gel (**m**).

Related to **Figure 1**.

Supplementary Figure 2

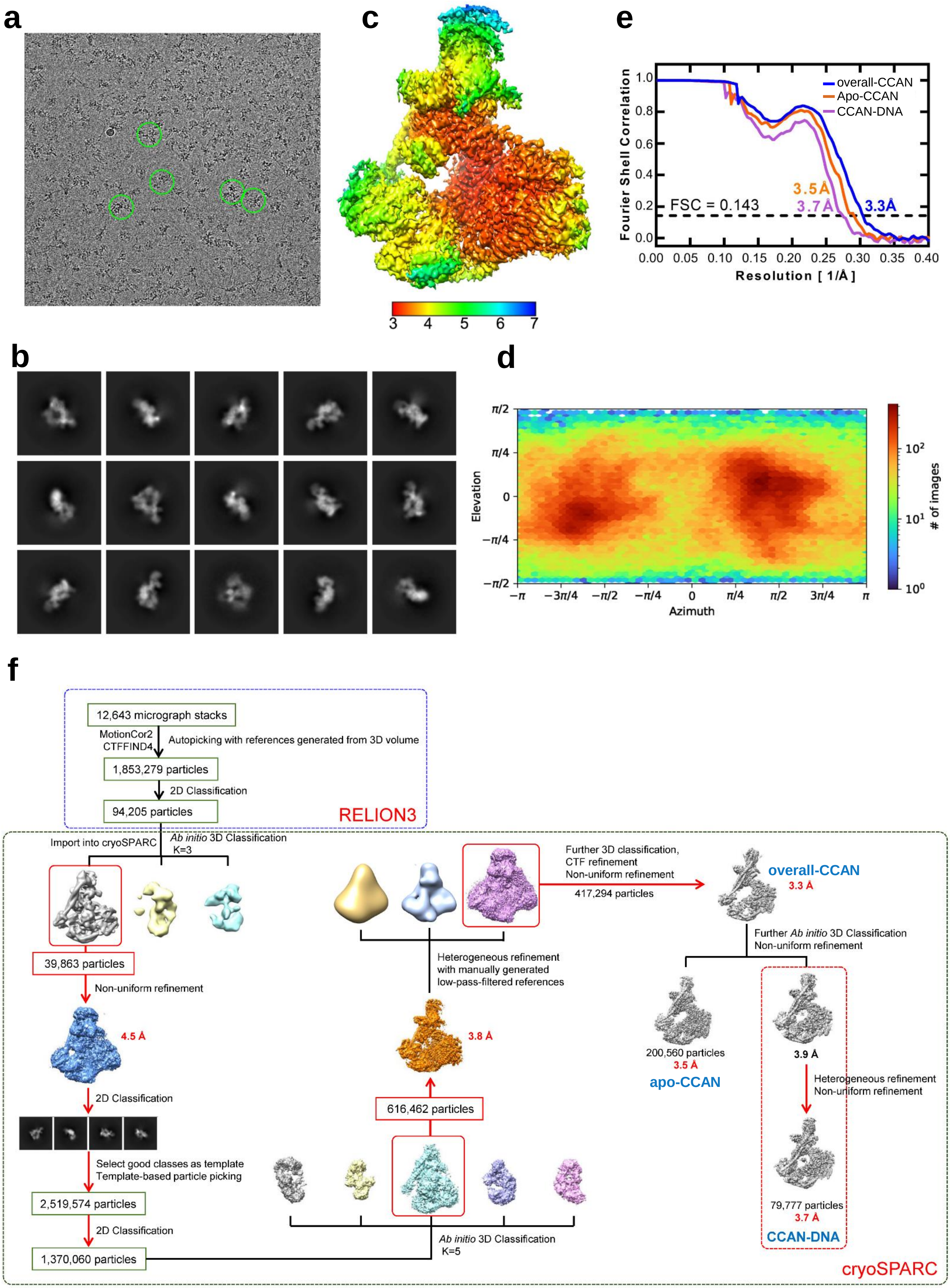

Supplementary Figure 2| Cryo-EM structure determination and validation of CCAN complex.
(**a**) A representative cryo-EM image of the CCAN complex. Typical particles are indicated in green circles.
(**b**) Typical two-dimensional classification images of the overall-CCAN complex.
(**c**) Local resolution estimation of the overall map by ResMap.
(**d**) Angular distribution of all particles in the final refinement for the overall map.
(**e**) FSC curves of the overall cryo-EM density map.overall-CCAN (blue), apo-CCAN (orange), and CCAN-DNA (violet).
(**f**) Flowchart of the data processing.
Related to **Figure 1**.

Supplementary Figure 3

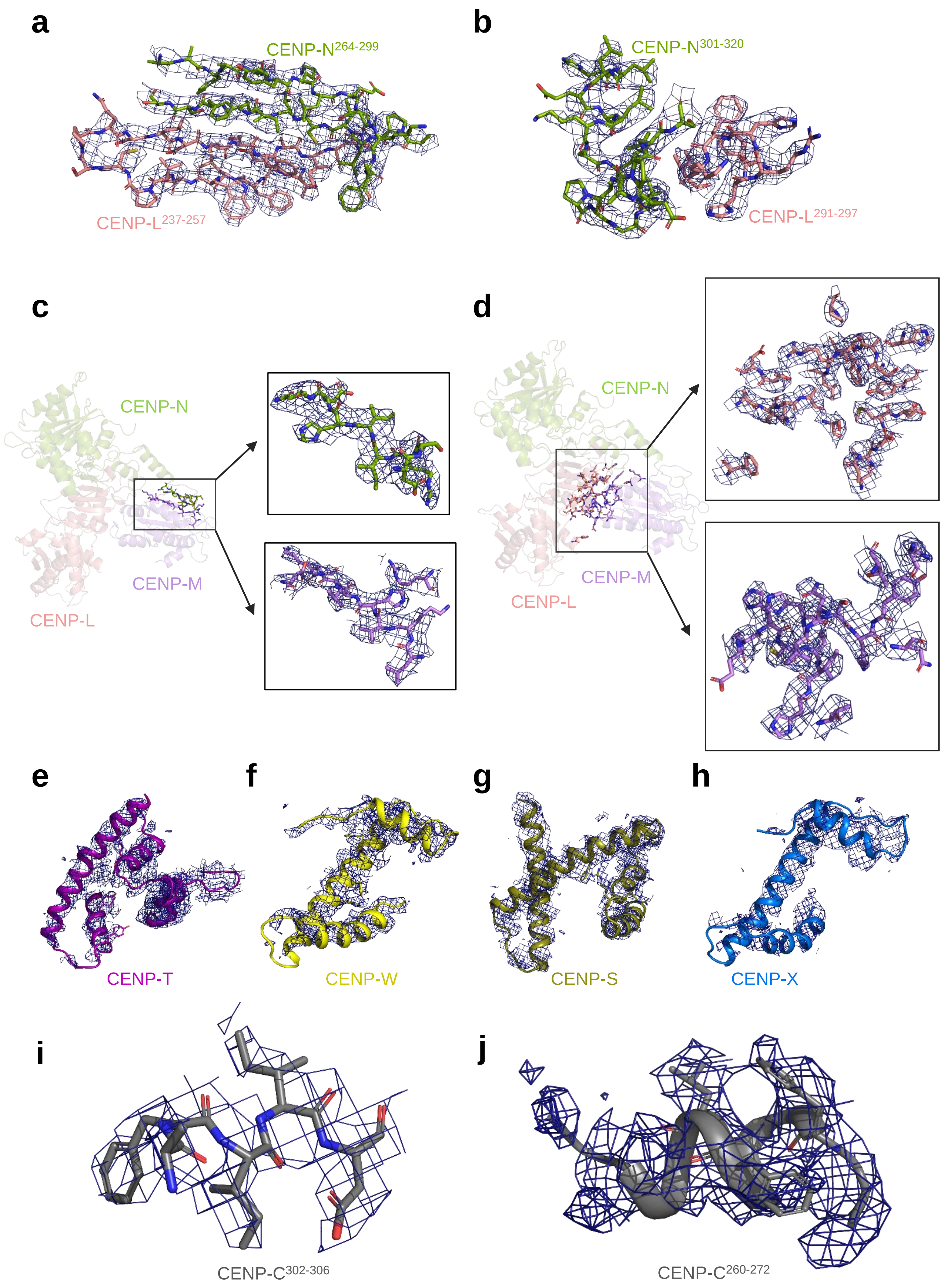

Supplementary Figure 3| Cryo-EM density map and structural model of CCAN.

(**a-b**) Cryo-EM map for the interface of CENP-LN. CENP-L and CENP-N interact through their C-termini. The interface among them can be divided into two parts, the first one is formed by their β-sheet toward the channel (**a**), and the other part is formed by α-helix and loop above the β-sheet (**b**). All the residues are shown as sticks and some potential phosphorylation sites are labeled by black box filled yellow.

(**c-d**) The binding interfaces between CENP-M and CENP-LN. A close-up view of the cryo-EM density maps in CENP-L/M (**c**) interface with CENP-L/N (**d**) boundary, indicated as black lines, are shown in right, respectively. Amino acid residues involved in interaction are shown as sticks.

(**e-h**) The density maps of CENP-T (**e**), CENP-W (**f**), CENP-S (**g**), CENP-X (**h**) show those of the subunits are clearly identified.

(**i-j**) The density maps of CENP-C$^{302-306}$ (**i**), CENP-C$^{260-272}$ **(j)** illustrate all the fragments clearly visualized.

Related to **Figure 1**.

Supplementary Figure 4

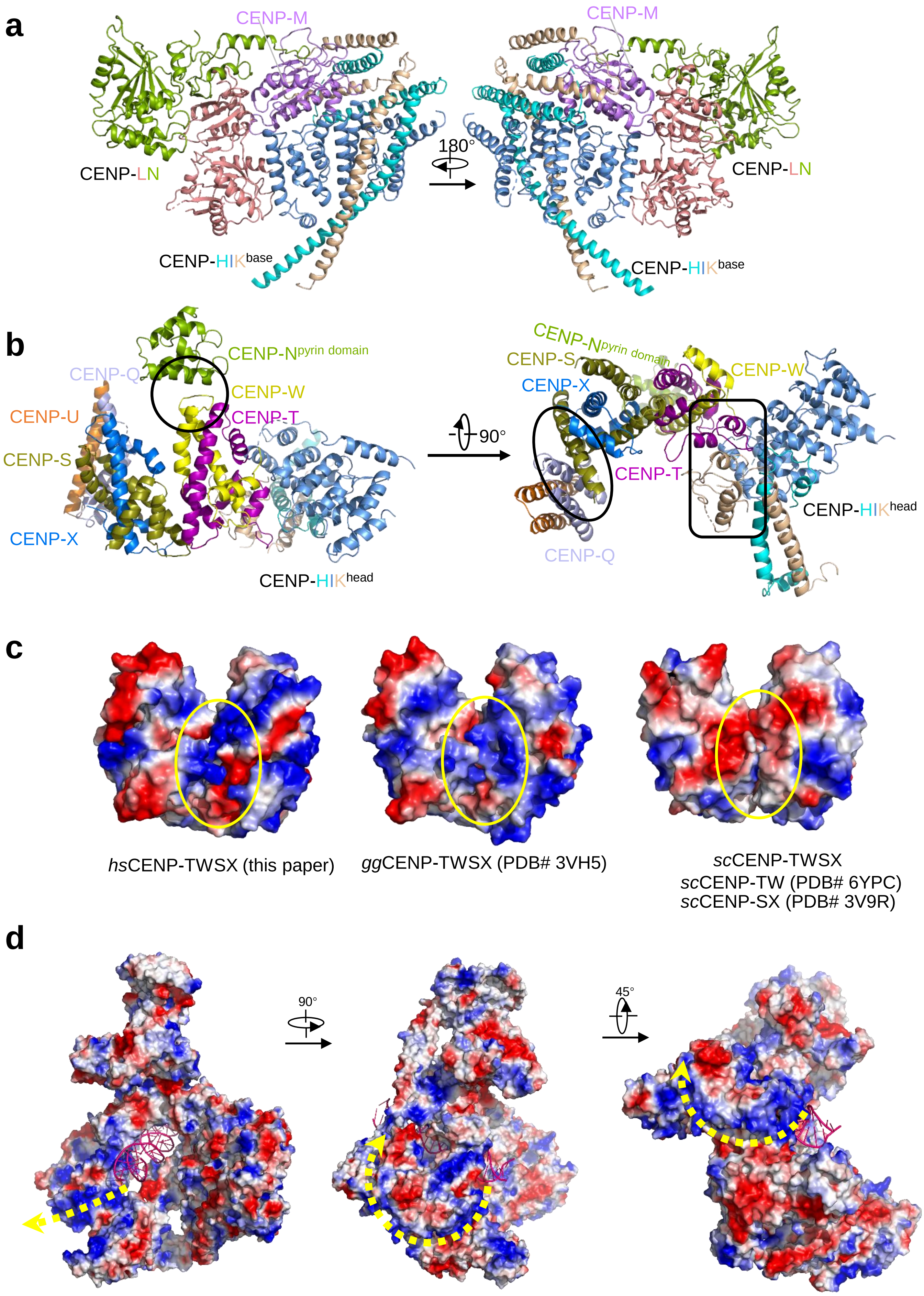

*hs*CENP-TWSX (this paper)

*gg*CENP-TWSX (PDB# 3VH5)

*sc*CENP-TWSX
*sc*CENP-TW (PDB# 6YPC)
*sc*CENP-SX (PDB# 3V9R)

Supplementary Figure 4| Properties and position of the CENP-M and CENP-TWSX.

(**a**) CENP-M binding site is located in the cavity generated by CENP-LN and CENP-HIK^{base}.

(**b**) The position of CENP-TWSX in CCAN complex. CENP-TWSX subcomplex is surrounded by CENP-N$^{\text{pyrin domain}}$, C-terminal of CENP-QU and CENP-HIK^{head}. The circle, ellipse and rounded rectangle represent the contact face of CENP-TWSX to CENP-N$^{\text{pyrin domain}}$, C-terminal of CENP-QU and CENP-HIK^{head}, respectively.

(**c**) Electrostatic surface annotation of CENP-TWSX sub-complex. The interfaces between CENP-TW and –SX are marked with yellow ellipse. In human and *G. gallus*, CENP-TW and CENP-SX connect with each other through the positively and negatively charge on their surfaces, whereas the positively region was replaced in budding yeast that result in CENP-SX unable to bind with CENP-TW. *G. gallus* (PDB code: 3VH5) and *S. cerevisiae* (CENP-TW from 6YPC, CENP-SX from 3V9R).

(**d**) Electrostatic potential surface depiction of CCAN complex. The yellow arc dot arrow illustrates that the DNA may bind continuous region of positive charge on the surface of CENP-TWSX to form nucleosome-like structure.

Related to **Figure 1**.

Supplementary Figure 5

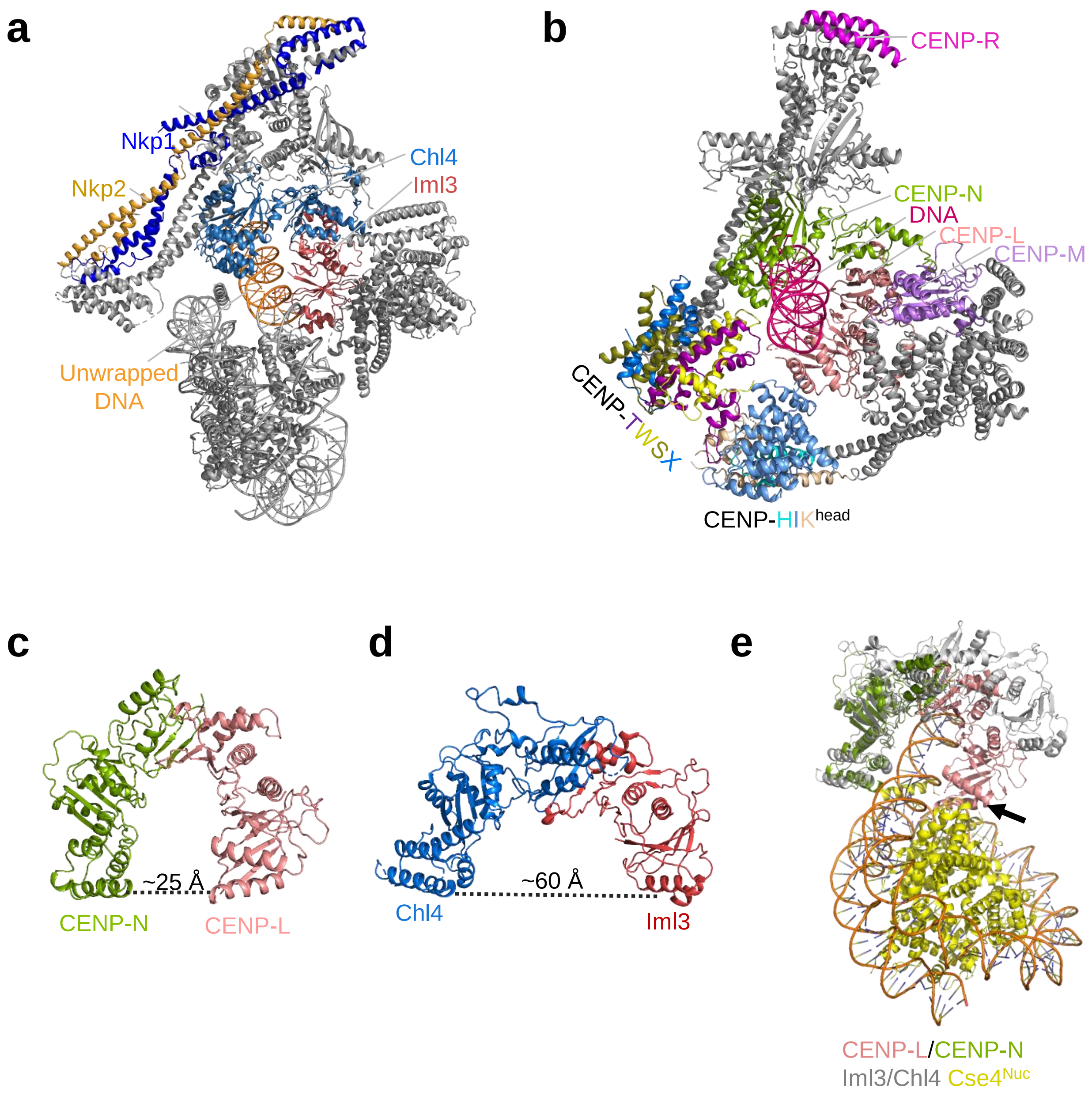

Supplementary Figure 5| Characteristics of human CENP-LN heterodimer for DNA or nucleosome binding.

(**a-b**) In *sc*CCAN-CENP-A^{Nuc} complex structure (**a**, PDB ID: 6QLD) and *hs*CCAN-DNA (**b**) showing DNA duplex engaged by the channel of the human CENP-LN subcomplex and the different subunits are labeled with distinct colors.

(**c-d**) The distance between human CENP-L and CENP-N, shown by black dot line, is ~25 Å (**c**) , but in budding yeast that is ~60 Å (**d**). The extend C-terminus of Chl4 causes a broader channel with Iml3 than its human counterpart, which explains why human CENP-LN is unable to interact with CENP-A nucleosome in the same conformation.

(**e**) Structure alignment of CENP-LN and Iml3^{CENP-L}/Chl4^{CENP-N}-Cse4^{CENP-A} nucleosome by superimpose CENP-N. Steric clash in CENP-L and *sc*Cse4 nucleosome is indicated by black arrow.

Related to **Figure 2**.

Supplementary Figure 6

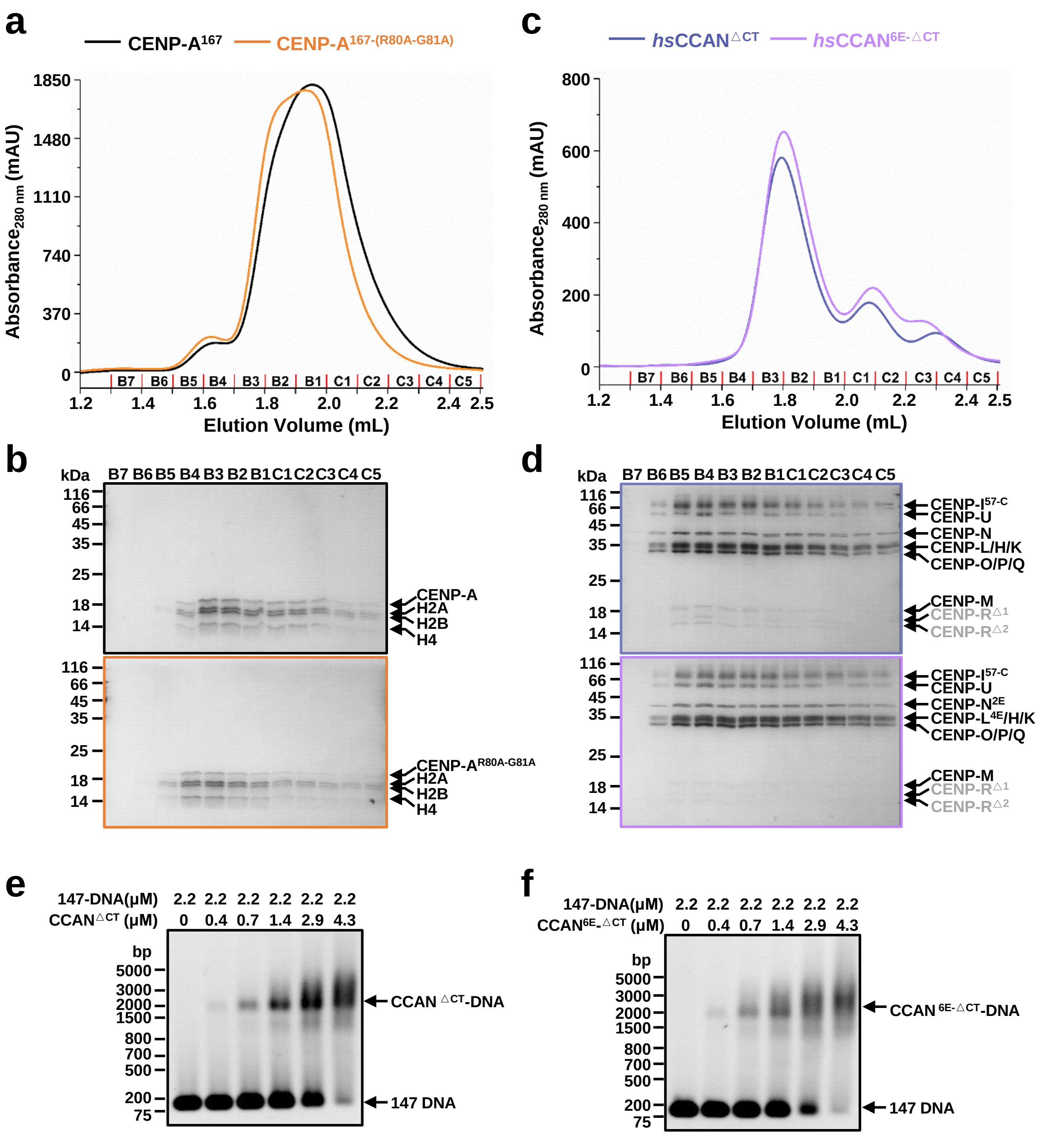

Supplementary Figure 6| The positively charged residues in CENP-LN channel are responsible for DNA binding.

(**a-b**) Elution profiles (**a**) of CENP-A^{167} and CENP-A$^{167-(R80A-G81A)}$ nucleosome in Superose 6 5/150 GL (GE health) and the Coomassie-blue stained 15% SDS-PAGE gel (**b**). Mutating the RG loop of CENP-A did not destabilize the CENP-A nucleosome.

(**c-d**) Elution profiles (**c**) of *hs*CCAN$^{\triangle CT}$ and *hs*CCAN$^{6E-\triangle CT}$ complex in Superose 6 5/150 GL (GE health) and the Coomassie-blue stained 15% SDS-PAGE gel (**d**). CENP-LN,CENP-HIKM and CENP-OPQUR formed stable complex. CENP-LN^{6E} mutant did not destabilize the *hs*CCAN$^{\triangle CT}$ complex.The intact CENP-R degrades into two parts named CENP-R $^{\triangle 1}$ and CENP-R $^{\triangle 2}$ which colored grey.

(**e-f**) EMSA assays tested the interaction between CCAN$^{\triangle CT}$ (**e**) and CCAN$^{6E-\triangle CT}$ (**f**) with 147 bp Widom 601 DNA.

Related to **Figure 2**.

Supplementary Figure 7

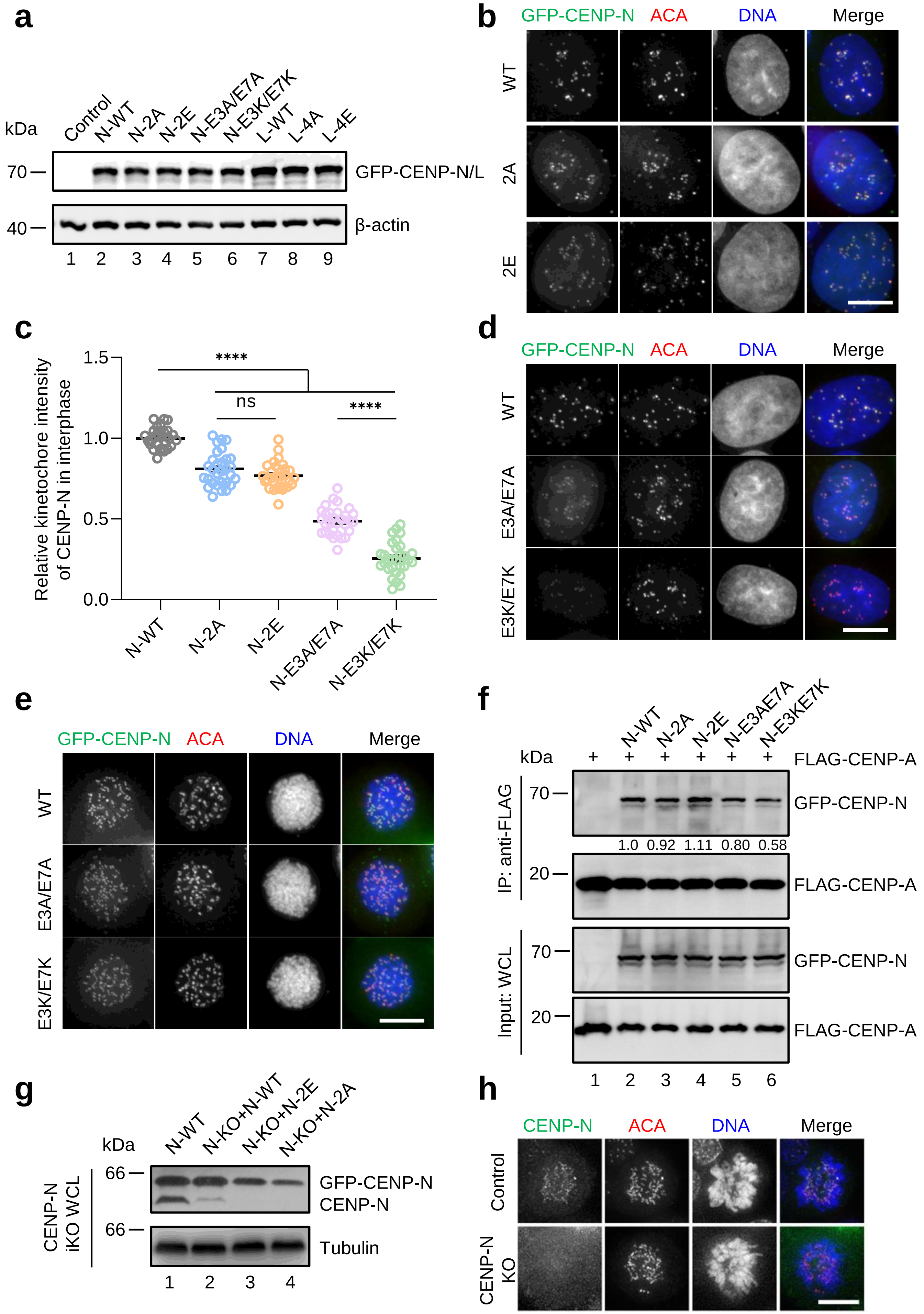

Supplementary Figure 7| CENP-A RG loop-binding is essential for localization of CENP-N to centromere of interphase cells.

(**a**) Characterization of expression level of HeLa cells stably expressing different GFP-CENP-N or GFP-CENP-L constructs as indicated. The cells were lysed and separated by SDS-PAGE. Then, samples were analyzed by Western blot using the indicated antibodies.

(**b**) Representative immunofluorescence images of interphase HeLa cells stably expressing GFP-CENP-N WT, K270A-K296A (2A), K270E-K296E (2E), respectively. Cells were fixed and co-stained for ACA (red) and DNA (blue). Scale bar, 10 µm.

(**c**) Statistical analysis of kinetochore intensity of various GFP-CENP-N mutants as treated in **b** and **d**. Bars represent the mean kinetochore intensity (±SEM) normalized to values of the CENP-N WT expressing group. Each dot represents one cell (30 cells from three independent experiments). Ordinary one-way ANOVA followed by Tukey's post hoc test was used to determine statistical significance. ****p < 0.0001; ns, not significant.

(**d**, **e**) Representative immunofluorescence images of interphase (**d**) HeLa cells or mitotic (**e**) HeLa cells stably expressing GFP-CENP-N WT, E3A/E7A, E3K/E7K, respectively. Cells were fixed and co-stained for ACA (red) and DNA (blue). To enrich mitotic cells, cells were treated with nocodazole for 2 hours. Then, cells were fixed and co-stained. Scale bar, 10 µm.

(**f**) CENP-N E3/E7 residues are required for effective association with CENP-A directly. FLAG-CENP-A and GFP-CENP-N, including wild type and various mutants, were transfected into HEK293T cells. 24 hours after transfection, cells were harvested and lysed. Immunoprecipitation assay was carried out using anti-FLAG M2 beads. Two hours after incubation at 4°C, the beads were washed 3 times and subjected to SDS-PAGE. The immunoprecipitates and inputs were analyzed by Western blotting using the indicated antibodies.

(**g**) Characterization of expression level of GFP-CENP-N wild type and mutants and CENP-N knockout efficiency in inducible CRISPR/Cas9-mediated CENP-N knockout (CENP-N iKO) HeLa cells. Doxycycline (1 µg/mL) was added to induce CENP-N knockout for 4 days. Cell lysates were probed with anti-CENP-N and tubulin antibodies.

(**h**) HeLa cells depleted of endogenous CENP-N were fixed and stained with CENP-N and ACA antibodies. CRISPR/Cas9-mediated endogenous CENP-N knockout was achieved by addition of Doxycycline (1 µg/mL). DNA was stained with DAPI. Scale bar, 10 µm.

Related to **Figure 3**.

Supplementary Figure 8

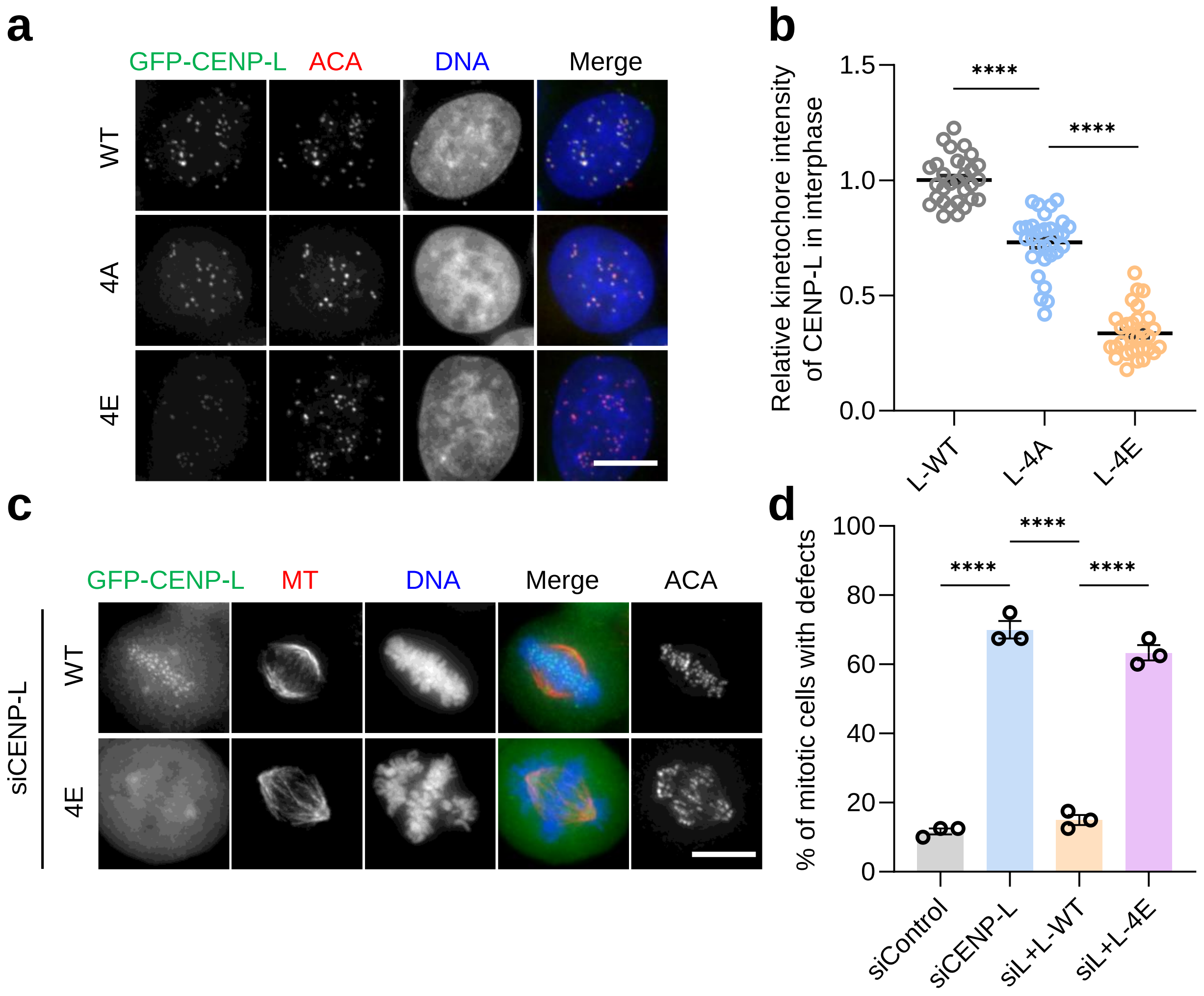

Supplementary Figure 8| DNA binding activity of CENP-L is critical for CENP-L kinetochore localization and faithful mitosis.

(**a**) Representative immunofluorescence images of interphase HeLa cells stably expressing GFP-CENP-L WT, K155A/R306A/K319A/K321A (4KA), K155E/R306E/K319E/K321E (4KE), respectively. Cells were fixed and co-stained for ACA (red) and DNA (blue). Scale bar, 10 µm.

(**b**) Statistical analysis of kinetochore intensity of various GFP-CENP-L mutants as treated in a. Bars represent the mean kinetochore intensity (±SEM) normalized to values of the CENP-L WT expressing group. Each dot represents one cell (30 cells from three independent experiments). Ordinary one-way ANOVA followed by Tukey's post hoc test was used to determine statistical significance. ****$p < 0.0001$.

(**c**) Representative immunofluorescence images of HeLa cells stably expressing siRNA-resistant GFP-CENP-L WT or 4KE treated with siCENP-L. 60 hours after siRNA transfection, cells were treated with MG132 for 2 hours and then fixed and co-stained for Tubulin (red), DNA (blue) and ACA (far-red). Scale bar, 10 µm.

(**d**) Bar graph representing the mean percentage of mitotic cells with defects (±SEM) of different groups of cells treated as in **c**. Data represent mean ± SEM (120 cells from three independent experiments). Ordinary one-way ANOVA followed by Tukey's post hoc test was used to determine statistical significance. ****$p < 0.0001$.

Related to **Figure 4**.

Supplementary Table 1. Cryo-EM data collection, refinement and validation statistics

	CCAN (EMDB-33197) (PDB 7XHO)	CCAN-DNA (EMDB-33196) (PDB 7XHN)
**Data collection and processing**		
Magnification	22,500	22,500
Voltage (kV)	300	300
Electron exposure (e–/Å^2)	50	50
Defocus range (μm)	-1.7~-2.7	-1.7~-2.7
Pixel size (Å)	1.22	1.22
Symmetry imposed	C1	C1
Initial particle images (no.)	1,853,279	1,853,279
Final particle images (no.)	200,560	79,777
Map resolution (Å)	3.29	3.71
FSC threshold	0.143	0.143
Map resolution range (Å)	2.4-30	2.4-30
**Refinement**		
Initial model used (PDB code)	*Ab initio*, 6EQT, 4P0T, 3VH5, I-TASSER, AF2	CCAN, 6QLD
Model resolution (Å)	4.3	4.7
FSC threshold	0.5	0.5
Model resolution range (Å)	2.4-30	2.4-30
Map sharpening *B* factor (Å^2)	-118.1	-113.7
Model composition		
Non-hydrogen atoms	22221	23246
Protein residues	2864	2864
Nucleotide	0	50
B factors (Å^2)		
Protein	86.61	69.81
Nucleotide		232.01
R.m.s. deviations		
Bond lengths (Å)	0.807	0.810
Bond angles (°)	0.004	0.004
Validation		
MolProbity score	2.26	2.28
Clashscore	15.08	16.28
Poor rotamers (%)	0.00	0.04
Ramachandran plot		
Favored (%)	92.26	92.50
Allowed (%)	7.60	7.33
Disallowed (%)	0.14	0.17
